# Supplementary material for: Preclinical Development of a Bacteriophage Cocktail for Treating Multidrug Resistant Pseudomonas aeruginosa Infections
Source: Microorganisms. 2021 Sep 21;9(9):2001. doi: 10.3390/microorganisms9092001 (PMC8464757; doi:10.3390/microorganisms9092001)
Supplement: Supplementary file 1 [file microorganisms-09-02001-s001.zip › microorganisms-1375145-supplementary.pdf]

Supplementary Materials

Table S1. Fifteen *P. aeruginosa* Bacteriophages Initial Titer.

Table 1. Titer (PFU mL^{-1}) of isolated phage stock solution

Phages	PA1	PA2	PA3	PA4	PA5	PA6	PA7	PA8	PA9	PA10	PA12	PA13	PA14	PA1[illegible]
Titer (PFU mL^{-1})	3.78 x 10^{8}	6.56 x 10^{8}	3.33 x 10^{8}	7.56 x 10^{9}	3.56 x 10^{9}	9.22 x 10^{8}	7.33 x 10^{6}	5.56 x 10^{10}	1.67 x 10^{10}	4.11 x 10^{10}	8.67 x 10^{9}	3.11 x 10^{10}	2.167 x 10^{10}	3.22 [illegible] 10^{1}[illegible]

Table S2. Antibiotic Resistance of Clinical Isolates. 9 CF and 11 non-CF clinical isolates were selected to determine the minimum inhibitory concentration (MIC) against 5 antibiotics (2 to 64 μg mL^{-1}) using 96-well plates in triplicates. Bacterial growth was measured by OD595 nm, n=3.

	**Antibiotic Minimum Inhibitory Concentration (μg mL^{-1})**				
***P. aeruginosa* Strain**	**Ciprofloxacin**	**Gentamicin**	**Imipenem**	**Netilmicin**	**Tobramycin**
**C450**	4	2	2	2	2
**C460**	16	64	2	64	16
**C461**	2	4	2	8	2
**C453**	2	8	4	16	2
**C458**	8	>64	>64	>64	>64
**C459**	4	32	8	64	8
**C462**	16	8	2	8	2
**C452**	64	4	2	8	4
**C457**	2	32	8	64	4
**C437**	2	16	4	32	2
**C420**	2	16	4	32	2
**C446**	2	16	4	4	16
**C410**	2	8	2	8	2
**C413**	2	16	4	16	2
**C438**	2	16	4	16	4
**C440**	2	8	2	16	2
**C433**	2	16	4	32	4
**C434**	2	4	2	2	2
**C389**	2	4	16	16	2
**C423**	16	64	8	>64	2

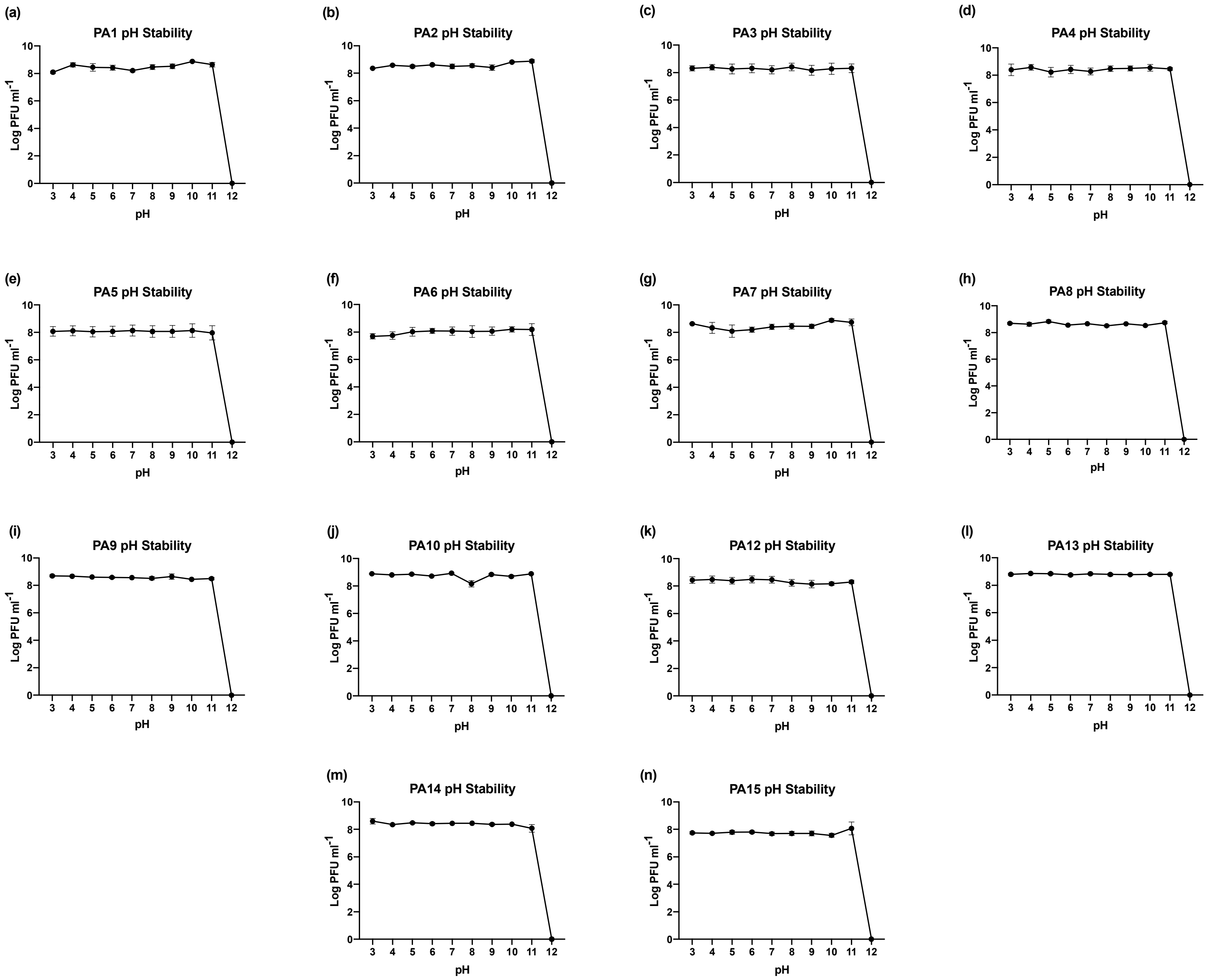
(a)
PA1 pH Stability
(b)
PA2 pH Stability
(c)
PA3 pH Stability
(d)
PA4 pH Stability
(e)
PA5 pH Stability
(f)
PA6 pH Stability
(g)
PA7 pH Stability
(h)
PA8 pH Stability
(i)
PA9 pH Stability
(j)
PA10 pH Stability
(k)
PA12 pH Stability
(l)
PA13 pH Stability
(m)
PA14 pH Stability
(n)
PA15 pH Stability
Log PFU ml-1
0
2
4
6
8
10
3
4
5
6
7
8
9
10
11
12
pH

Figure S1. Bacteriophage pH Stability Test. Phages were incubated at different pH (3 to 12) conditions with phage titer determined to investigate their stability. Data expressed as mean ± SEM for three independent experiments. Significant difference compared to values at pH=7 was determined using One-way ANOVA. ****, $p<0.0001$.

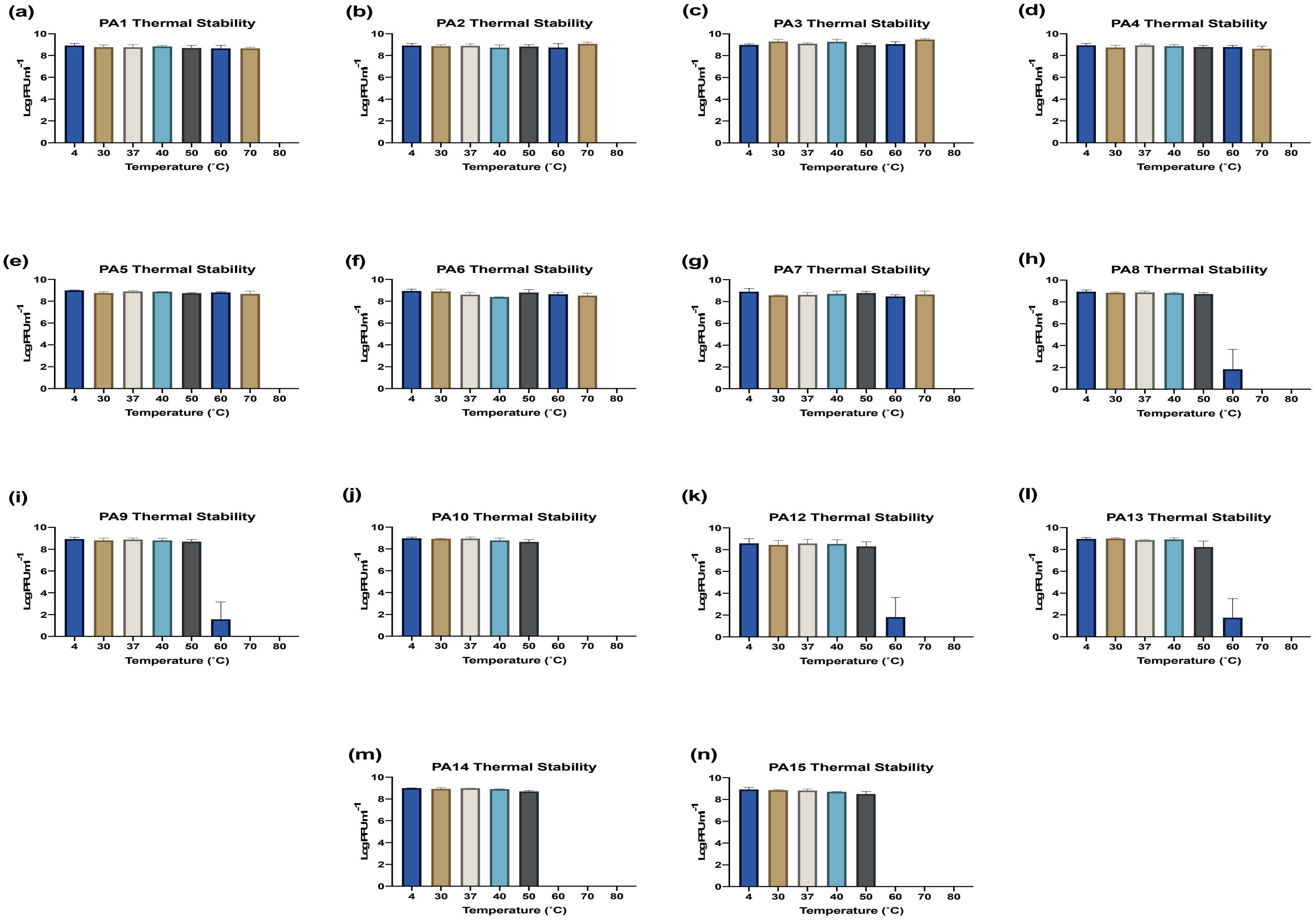
(a)
PA1 Thermal Stability
(b)
PA2 Thermal Stability
(c)
PA3 Thermal Stability
(d)
PA4 Thermal Stability
(e)
PA5 Thermal Stability
(f)
PA6 Thermal Stability
(g)
PA7 Thermal Stability
(h)
PA8 Thermal Stability
(i)
PA9 Thermal Stability
(j)
PA10 Thermal Stability
(k)
PA12 Thermal Stability
(l)
PA13 Thermal Stability
(m)
PA14 Thermal Stability
(n)
PA15 Thermal Stability
LogPFUml-1
0 2 4 6 8 10
4 30 37 40 50 60 70 80
Temperature (˚C)

Figure S2. Bacteriophage Thermal Stability Test. Stability measured by viability in PFU ml^{-1} for phages incubated in temperature conditions ranging between 4˚C-80˚C for 1 hour. Data expressed as mean ± SEM for three independent experiments. Significant difference compared to 4˚C was determined using One-way ANOVA. ****, $p<0.0001$.

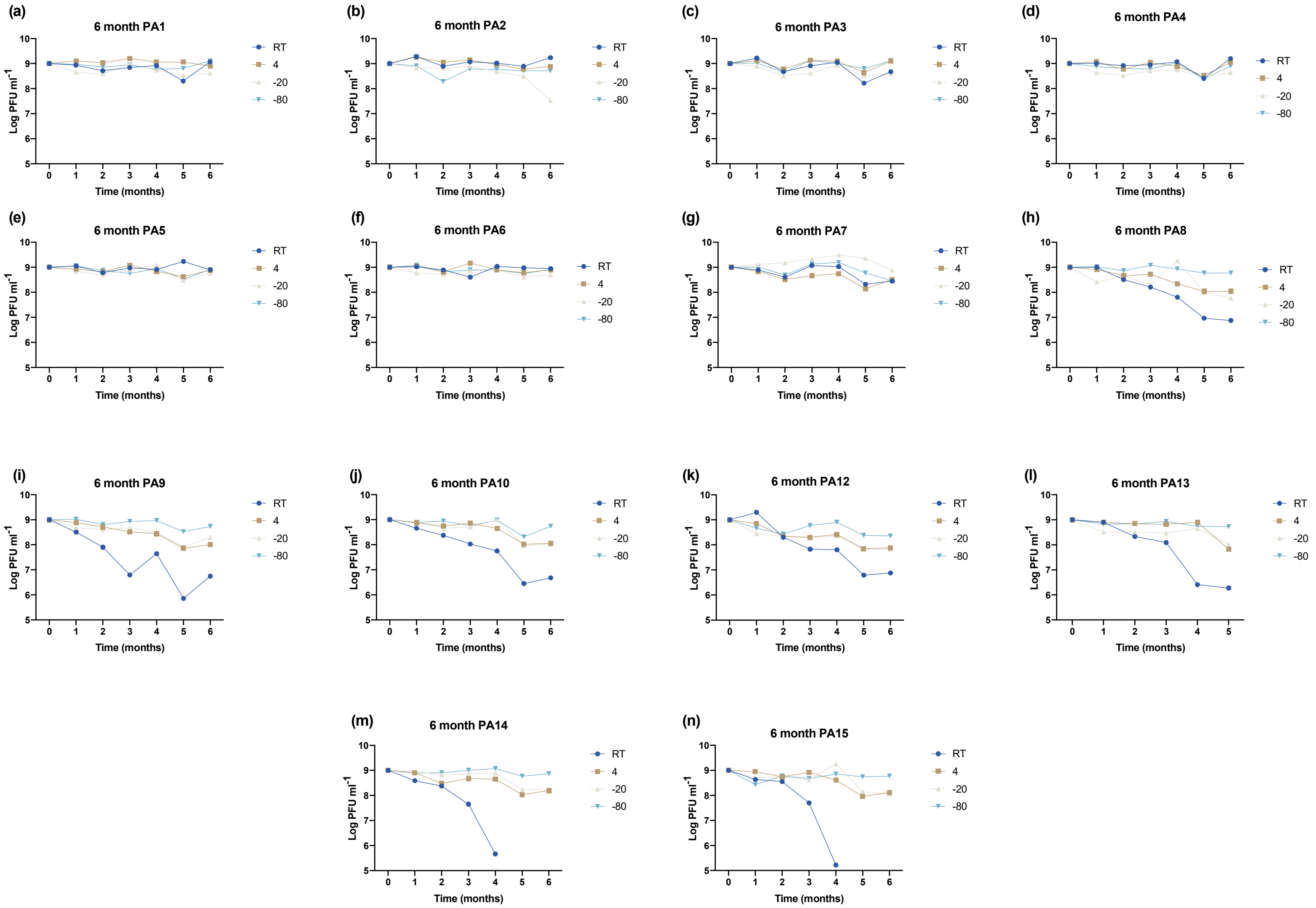

Figure S3. Bacteriophage Long-term Storage Stability Test. 6-month long-term stability of phages stored at room temperature (RT), 4˚C, -20˚C and -80˚C. Data expressed as mean.

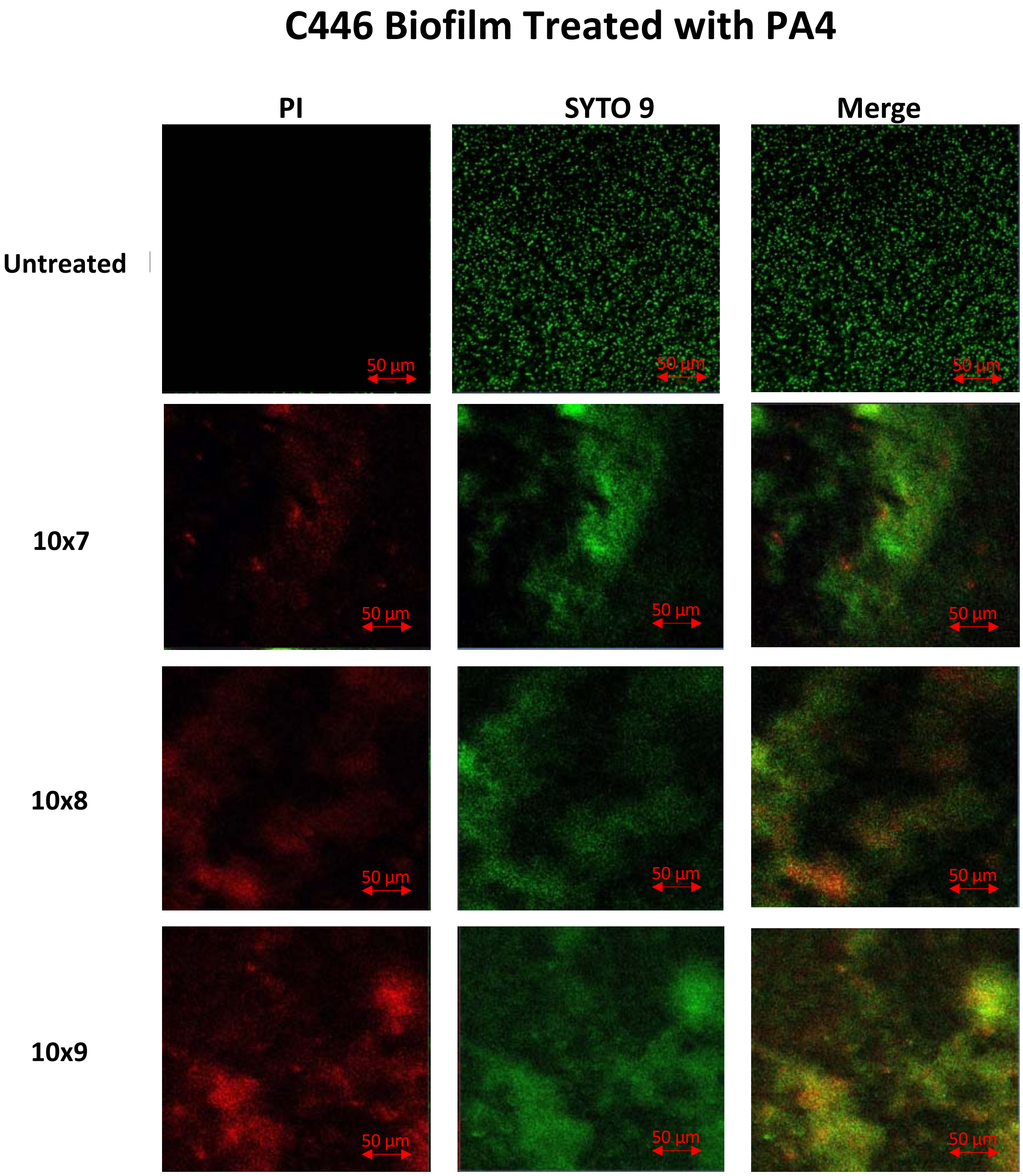

Figure S4. a) C446 biofilm treated with PA4.

C446 biofilm treated with PA4 at 10x7, 10x8 and 10x9 PFU mL^{-1} respectively. Biofilms without treatment (untreated) were used as control. LIVE/DEAD® Bacterial Viability Kit was used showing dead (PI, red) and live (SYTO® 9, green) biofilm associated bacteria in samples representative of each treatment group.

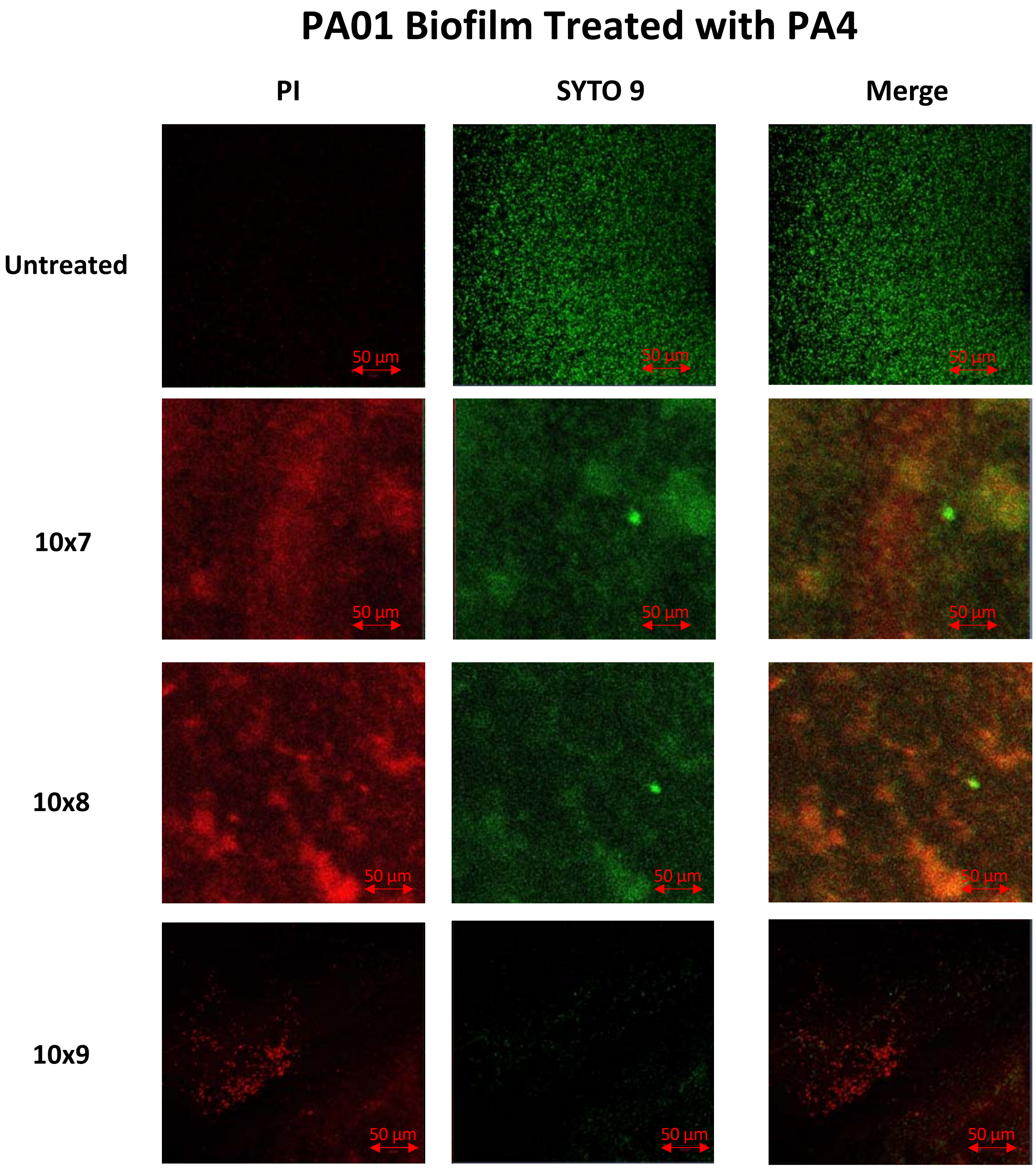

Figure S4. b) PA01 biofilm treated with PA4.

PA01 biofilm treated with PA4 at 10x7, 10x8 and 10x9 PFU mL^{-1} respectively. Biofilms without treatment (untreated) were used as control. LIVE/DEAD® Bacterial Viability Kit was used showing dead (PI, red) and live (SYTO® 9, green) biofilm associated bacteria in samples representative of each treatment group.

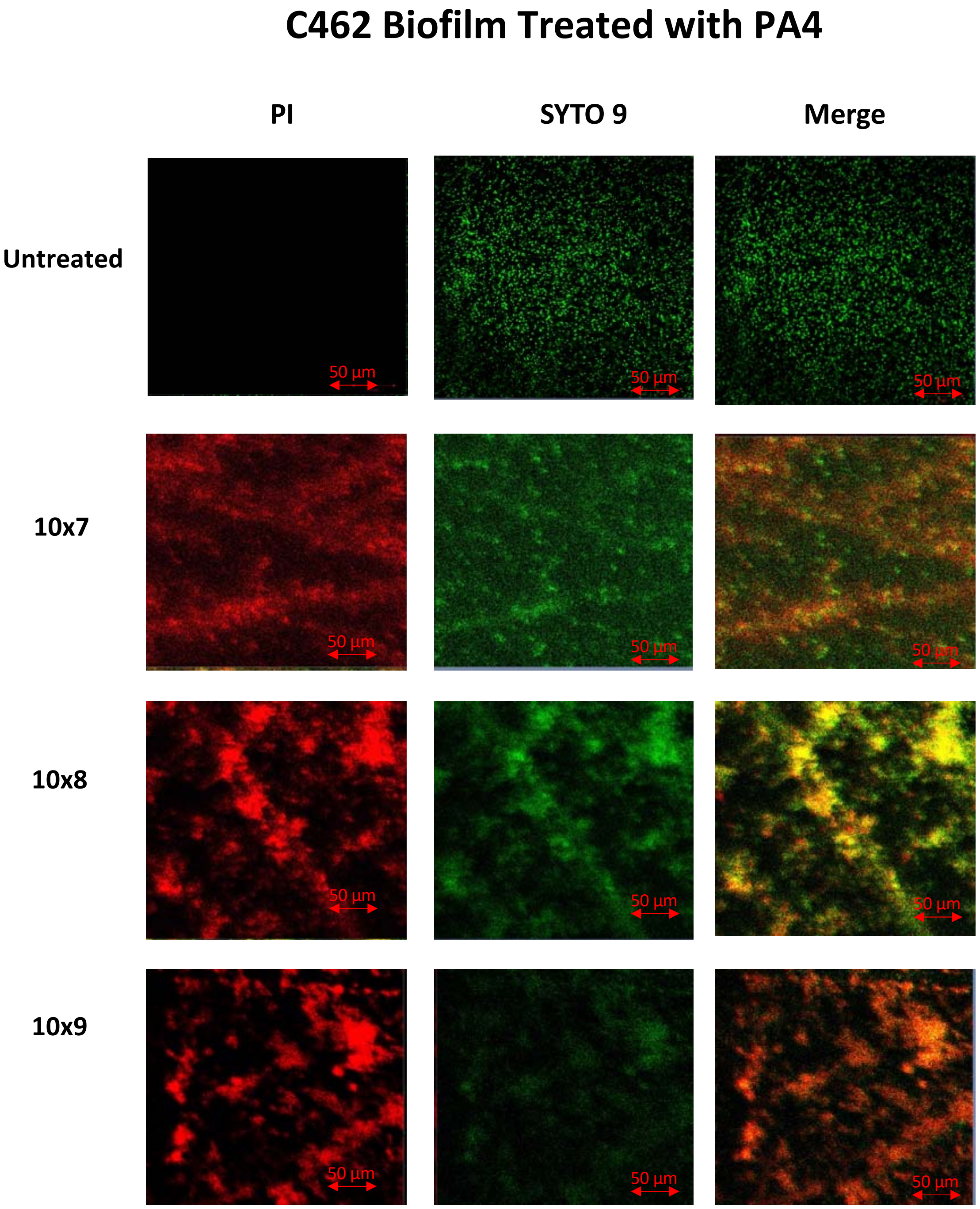

Figure S4. c) C462 biofilm treated with PA4.

C462 biofilm treated with PA4 at 10x7, 10x8 and 10x9 PFU mL^{-1} respectively. Biofilms without treatment (untreated) were used as control. LIVE/DEAD® Bacterial Viability Kit was used showing dead (PI, red) and live (SYTO® 9, green) biofilm associated bacteria in samples representative of each treatment group.
